# Single shot polarization resolved coded aperture imaging

**DOI:** 10.1038/s41598-025-04657-2

**Published:** 2025-07-02

**Authors:** Narmada Joshi, Vipin Tiwari, Aile Tamm, Joseph Rosen, Vijayakumar Anand

**Affiliations:** 1 Institute of Physics, University of Tartu, W. Ostwaldi 1, 50411 Tartu, Estonia; 2 School of Electrical and Computer Engineering, Ben Gurion University of the Negev, P.O. Box 653, 8410501 Beer-Sheva, Israel; 3 Optical Sciences Center, Swinburne University of Technology, Hawthorn, Melbourne, VIC 3122 Australia

**Keywords:** Optics and photonics, Optical physics

## Abstract

Coded aperture imaging (CAI) is a well-established indirect imaging technique consisting of two steps, namely optical recording and computational reconstruction. In the recent years, CAI technique has been extended to image along 3D space and also the spectrum with a single camera shot. In this study, we developed CAI for 3D imaging along 2D space and polarization using dual orthogonal polarization phase-modulation (DOPP) technique. DOPP-CAI has been demonstrated for 3D imaging with only one birefringent optical modulator and without any polarization sensitive image sensors. The theory, simulation and proof-of-concept experimental results are presented. The results demonstrate a one-to-one unique intensity-polarization mapping owing to a significant polarization discriminated blur in CAI. We believe that the developed DOPP-CAI can benefit multimodal imaging, birefringent imaging, holography and microscopy.

## Introduction

Coded aperture imaging (CAI) is a powerful lensless imaging technique based on the two-step imaging process, i.e., optical recording and computational reconstruction^1,2^. The first step is an optical process, where the point spread function (PSF) of the system is recorded by mounting a coded mask between a point object and an image sensor and then under identical recording conditions as those for recording the PSF, the response for an object intensity (ROI) is recorded. The second step is a computational process, where the recorded PSF and ROI are processed in the computer to reconstruct the image of the object. This approach allows the use of different thin binary masks with only two levels, either an amplitude version with transmittance values of 0 and 1 or phase version with phase height of 0 and π, for performing imaging instead of a lens. The motivation for the development of CAI originated from the lack of advanced manufacturing technologies for manufacturing a lens for non-visible areas of the electromagnetic spectrum, such as X-rays and Gamma rays^1,2^. Therefore, with CAI, lenses can be replaced by thin binary diffractive elements, which are easy to manufacture, and by a computational reconstruction process, the image can be reconstructed. The above advantages of CAI made it a robust imaging technique for various applications in the non-visible regions of the electromagnetic spectra. Moreover, CAI was also adapted quickly to the visible region of imaging due to its exceptional imaging capabilities.

The development of CAI made a significant impact on the course of development of imaging technologies in general^3–12^. CAI techniques developed along two main directions namely design of coded masks^13^ and computational reconstruction methods^14^, both directions to enhance the signal to noise ratio (SNR) of image reconstruction. Some widely used coded masks are the uniformly redundant array (URA)^15^, modified URA (MURA)^16^, Fresnel zone aperture (FZA)^17^ and scattering mask (SM)^1,2,18,19^. In computational reconstruction methods, some important developments include the development of a phase only filter^20^, the Lucy-Richardson algorithm^21,22^, Weiner deconvolution^23^, non-linear reconstruction^24^, Lucy-Richardson-Rosen algorithm (LRRA)^25^, adaptive and non-iterative algorithms^26,27^, deep learning-based reconstruction^28^, and incoherent non-linear deconvolution with an iterative algorithm (INDIA)^14^.

In the recent years, there has been an increase in the number of researches on CAI focused on increasing the number of imaging dimensions achieved within a few camera recordings. Some notable works in this direction are the interferenceless coded aperture correlation holography (I-COACH)^29^, diffuser microscope^30^, diffuser CAM^31^ and spectral imaging using a diffuser^32,33^. In all the above studies, the PSF was recorded for the additional dimensions, assuming a unique response for the different dimensions. By processing the ROI with the PSF library of a certain dimension, the object information variation along that dimension can be reconstructed. There are also researches on spectrum and polarization imaging using coded apertures^34–36^. There are some interesting studies on phase imaging with polarization sensitive device^37^ and polarization coded aperture^38^ but they are not useful for extracting polarization specific information.

Incoherent digital holography techniques have been developed for 4D imaging along 3D space and polarization by Tahara^39–41^ in the last few years. However, the above techniques require at least two spatial light modulators (SLMs) with complicated optical configurations, several camera shots and can reconstruct images only along two orthogonal polarization states. Recently, an I-COACH based 5D imaging technique has been developed using one SLM and one binary amplitude-only diffractive optical element fabricated using lithography technique and demonstrated using a single camera shot^42^. The above methods^39–42^, even though offer 5D imaging capabilities, the cost of achieving this with two SLMs and many numbers of camera shots formed as obstacles preventing their broader applicability in other areas.

In this study, we introduce a 3D imaging technique along 2D space and polarization using a single SLM and a single camera shot called as dual orthogonal polarization phase-modulation based coded aperture imaging (DOPP-CAI) for the first time. We developed a unique optical configuration that allows us perform two phase modulations at orthogonal polarization orientations with the same SLM^43,44^. Therefore, a one-to-one unique intensity-polarization state map can be created allowing to discriminate different polarization states clearly. Simulation studies and proof of concept experimental studies have been carried out. We proposed that the developed technique has the potential to be easily extended to spectrum and depth, enabling up to 6D imaging in the near future. The manuscript consists of five sections. The methodology is presented in the next section. The experiments are discussed in the third section. The results are presented and discussed in the fourth section. The final section discusses the conclusion and future perspectives of DOPP-CAI.

## Methodology

**Fig. 1:**
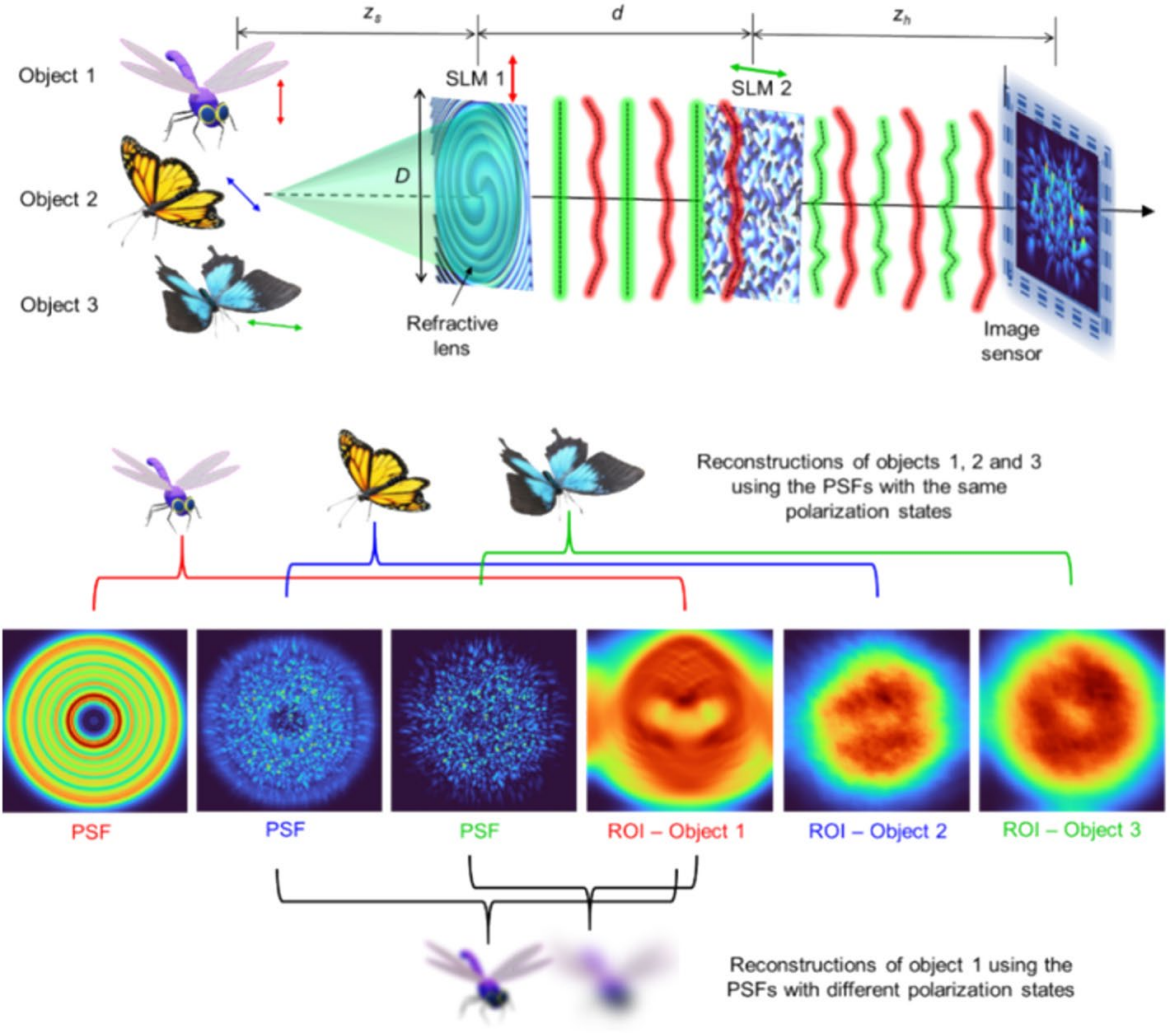
Optical configuration of dual orthogonal polarization phase-modulation CAI (DOPP) and imaging process. (*PSF* point spread function, *ROI* response for an object intensity).

The proposed DOPP-CAI consists of two components, namely the dual orthogonal polarization phase-modulation process and imaging process. The optical configuration of DOPP-CAI is shown in (Fig. 1). Light from an object point is incident on a refractive lens located at a distance of *z*_*s*_ and then on two SLMs, SLM1 and SLM2 separated by a distance of *d* with their active axes orthogonal to one another. The light modulated by the two SLMs are recorded by an image sensor located at a distance of *z*_*h*_ from SLM2. The complex amplitude before the refractive lens is given as $\sqrt {I_{s} } C_{1} L\left( {\frac{{\overline{r}_{s} }}{{z_{s} }}} \right)Q\left( {\frac{1}{{z_{s} }}} \right)$, where $\sqrt{{I}_{s}}$ is the amplitude of the point object, $L\left(\frac{\overline{s}}{z }\right)=\mathit{exp}\left[i2\pi {\left(\lambda z\right)}^{-1}\left({s}_{x}x+{s}_{y}y\right)\right]$ is the linear phase function, $Q(a)=\mathit{exp}\left[i\pi a{\lambda }^{-1}\left({x}^{2}+{y}^{2}\right)\right]$ is the quadratic phase function, *C*_1_ is a complex constant and λ is the wavelength. The theoretical analysis assumes that the object is illuminated by a quasi-monochromatic, spatially incoherent and temporally coherent illumination. Considering that the refractive lens has a focal length of *f*, the complex amplitude after the refractive lens is $\sqrt {I_{s} } C_{1} L\left( {\frac{{\overline{r}_{s} }}{{z_{s} }}} \right)Q\left( {\frac{1}{{z_{s} }}} \right)Q\left( { - \frac{1}{f}} \right)$. An SLM (SLM1) is present in tandem with the refractive lens with its active axis parallel to the plane of the paper. On SLM1, a coded mask with a phase distribution of a spiral lens (SL) given as $exp\left({j\Phi }_{SL}\right)=\mathit{exp}\left[-i\pi ({\lambda {f}_{1})}^{-1}{R}^{2}\right]\times exp\left(-iL\theta \right)$, where $R={\left({x}^{2}+{y}^{2}\right)}^{1/2}$ is displayed, where *L* is the topological charge, assuming

$$f = z_{s}, \quad f_{1} = \frac{d}{2}, \quad f_{2} = \left( \frac{2}{d} + \frac{1}{z_{h}} \right)^{-1}$$

*f*_1_ is the focal length of the SL and *f*_2_ is the focal length of the QRDL. In the concept figure (Fig. 1), *L* = 3, the complex amplitude after the SLM1 is given as $\it \it \sqrt {I_{s} } C_{1} L\left( {\frac{{\overline{r}_{s} }}{{z_{s} }}} \right)Q\left( {\frac{1}{{z_{s} }}} \right)Q\left( { - \frac{1}{f}} \right)\textit{exp} \left( {j\Phi_{SL} } \right)$ which is propagated by a distance of *d* before it is incident on the next SLM (SLM2) whose active axis is oriented orthogonal to the active axis of the SLM1 such that it is perpendicular to the plane of the paper. The complex amplitude just before the SLM2 is given as $\sqrt {I_{s} } C_{1} L\left( {\frac{{\overline{r}_{s} }}{{z_{s} }}} \right)Q\left( {\frac{1}{{z_{s} }}} \right)Q\left( { - \frac{1}{f}} \right)\textit{exp} \left( {j\Phi_{SL} } \right) \otimes Q\left( \frac{1}{d} \right)$, where ‘$\otimes$’ is a 2D convolutional operator. On the SLM2, another coded mask, phase of a quasi-random lens with a phase distribution $exp\left({j\Phi }_{QRL}\right)=\mathit{exp}\left[-i\pi ({\lambda {f}_{2})}^{-1}{R}^{2}\right]\times exp\left(-i{\Phi }_{r}\right)$, where ${\Phi }_{r}$ is a 2D quasi-random distribution [0, 2π] whose scattering ratio σ can be controlled by the Gerchberg-Saxton algorithm (GSA) by applying a limited support mask constraint in the spectral domain^45,46^. The complex amplitude after the SLM2 is given as $\left\{ {\sqrt {I_{s} } C_{1} L\left( {\frac{{\overline{r}_{s} }}{{z_{s} }}} \right)Q\left( {\frac{1}{{z_{s} }}} \right)Q\left( { - \frac{1}{f}} \right)\textit{exp} \left( {j\Phi_{SL} } \right) \otimes Q\left( \frac{1}{d} \right)} \right\}\textit{exp} \left( {j\Phi_{QRL} } \right)$. The light modulated by the SLM2 reaches the image sensor located at a distance of *z*_*h*_ from the SLM2 and recorded. The recorded intensity distribution is given as $I_{PSF} = \left| {\left\{ {\sqrt {I_{s} } C_{1} L\left( {\frac{{\overline{r}_{s} }}{{z_{s} }}} \right)Q\left( {\frac{1}{{z_{s} }}} \right)Q\left( { - \frac{1}{f}} \right)\textit{exp} \left( {j\Phi_{SL} } \right) \otimes Q\left( \frac{1}{d} \right)} \right\}\textit{exp} \left( {j\Phi_{QRL} } \right) \otimes Q\left( {\frac{1}{{z_{h} }}} \right)} \right|^{2}$. The above calculation did not take into consideration the polarization of the point object.

Depending upon the polarization orientation of the light from the point object, there are different possible intensity distributions recorded by the image sensor. Let us consider three orientations: (a) φ = 0, polarized along the active axis of the SLM1, (b) φ = π/2, polarized along the active axis of the SLM2, (c) φ = π/4, polarized at ± π/4 with respect to the active axes of the SLM1 and SLM2. When φ = 0, the light from the point object is modulated by the coded mask displayed on the SLM1, but upon reaching SLM2, there is no modulation as the active axis is orthogonal to the polarization state of the incoming light. The recorded intensity distribution when φ = 0 is given as.

$I_{PSF} \left( {\varphi = 0} \right) = \left| {\left\{ {\sqrt {I_{s} } C_{1} L\left( {\frac{{\overline{r}_{s} }}{{z_{s} }}} \right)Q\left( {\frac{1}{{z_{s} }}} \right)Q\left( { - \frac{1}{f}} \right)\textit{exp} \left( {j\Phi_{SL} } \right) \otimes Q\left( \frac{1}{d} \right)} \right\} \otimes Q\left( {\frac{1}{{z_{h} }}} \right)} \right|^{2}$ Which can be simplified as

1 $$\it \it \it \it \it \it \it I_{PSF} \left( {\varphi = 0} \right) = \left| {\sqrt {I_{s} } C_{1} L\left( {\frac{{\overline{r}_{s} }}{{z_{s} }}} \right)Q\left( {\frac{1}{{z_{s} }}} \right)Q\left( { - \frac{1}{f}} \right)\textit{exp} \left( {j\Phi_{SL} } \right) \otimes Q\left( {\frac{1}{{d + z_{h} }}} \right)} \right|^{2} .$$

When φ = π/2, the light from the point object is not modulated by the coded mask displayed on the SLM1 but only by the coded mask displayed on the SLM2. The recorded intensity distribution is given as

2 $$I_{PSF} \left( {\varphi = \frac{\pi }{2}} \right) = \left| {\left\{ {\sqrt {I_{s} } C_{1} L\left( {\frac{{\overline{r}_{s} }}{{z_{s} }}} \right)Q\left( {\frac{1}{{z_{s} }}} \right)Q\left( { - \frac{1}{f}} \right) \otimes Q\left( \frac{1}{d} \right)} \right\}\textit{exp} \left( {j\Phi_{QRL} } \right) \otimes Q\left( {\frac{1}{{z_{h} }}} \right)} \right|^{2} .$$

The third case φ = π/4 has an interesting outcome. Nearly 50%—φ = 0 component from the point object is modulated by the coded mask displayed on the SLM1 and the remaining 50%—φ = π/2 component is not modulated. When this mixture of light is incident on the SLM2, the part modulated by the SLM1 has an orthogonal polarization with respect to the active axis of SLM2 and so it is not modulated at the SLM2. However, the part that is not modulated by the SLM1 has a polarization orientation aligned with the active axis of the SLM2 and so it is modulated by the coded mask displayed on the SLM2. Therefore, after SLM2, the light has two orthogonal components that are modulated by SLM1 only and modulated by SLM2 only. Since, the two components are orthogonal to each other, they do not interfere but their intensities add up at the image sensor. Therefore, the recorded intensity distribution is given as

3 $$\begin{aligned} I_{PSF} \left( {\varphi = \frac{\pi }{4}} \right) & = \left| {\left\{ {\sqrt {I_{s} } C_{1} L\left( {\frac{{\overline{r}_{s} }}{{z_{s} }}} \right)Q\left( {\frac{1}{{z_{s} }}} \right)Q\left( { - \frac{1}{f}} \right) \otimes Q\left( \frac{1}{d} \right)} \right\}\textit{exp} \left( {j\Phi_{QRL} } \right) \otimes Q\left( {\frac{1}{{z_{h} }}} \right)} \right|^{2} \\ & + \left| {\sqrt {I_{s} } C_{1} L\left( {\frac{{\overline{r}_{s} }}{{z_{s} }}} \right)Q\left( {\frac{1}{{z_{s} }}} \right)Q\left( { - \frac{1}{f}} \right)\textit{exp} \left( {j\Phi_{SL} } \right) \otimes Q\left( {\frac{1}{{d + z_{h} }}} \right)} \right|^{2} . \\ \end{aligned}$$

The proposed DOPP-CAI is a linear, shift-invariant system with a magnification *M* = *z*_*h*_/*z*_*s*_ and so the point spread function can be expressed as

4 $$I_{PSF} \left( {\overline{r}_{0} ;\overline{r}_{s} ,z_{s} ,\varphi } \right) = I_{PSF} \left( {\overline{r}_{0} - \frac{{z_{h} }}{{z_{s} }}\overline{r}_{s} ;0,z_{s} ,\varphi } \right).$$

A 2D object *O* consisting of *N* points when placed in the location of the point object, it will generate *N* shifted *I*_*PSF*_s, which are algebraically summed at the image sensor. The object *O* can be mathematically expressed as a collection of Delta functions given as

5 $$\mathit{O}\left( {\overline{r}_{s} } \right) = \sum\nolimits_{P}^{N} {a_{p} \delta } \left( {\overline{r} - \overline{r}_{s,p} } \right).$$

The intensity distribution recorded for the 2D object at the image sensor is given as

6 $$I_{O} \left( {\overline{r}_{0} ;z_{s} ,\varphi } \right) = \sum\nolimits_{P}^{N} {a_{p} I_{PSF} } \left( {\overline{r}_{0} - \frac{{z_{h} }}{{z_{s} }}\overline{r}_{s,p} ;0,z_{s} ,\varphi } \right).$$

From *I*_*O*_ and *I*_*PSF*_, the information of *O* can be obtained using one of the reconstruction methods or deconvolution methods^14,20–28^. This can be expressed as $I_{R} = I_{O} \odot I_{PSF}$, where ‘$\odot$’ denotes the reconstruction operation. The goal of the study is to achieve polarization dependent blur. In I-COACH, depth-dependent blur was demonstrated^29–31^. Here, comparing, Eqs. (1)-(3), it can be clearly observed that the cross-correlation value is smaller than the auto-correlation value which ensures the polarization dependent blur during reconstruction of polarized *O* by processing ${I}_{O}$ using the PSF library. The two coded masks, namely spiral lens and quasi-random lens given here are only examples and the two coded masks can be anything depending upon the photon budget requirements.

**Fig. 2:**
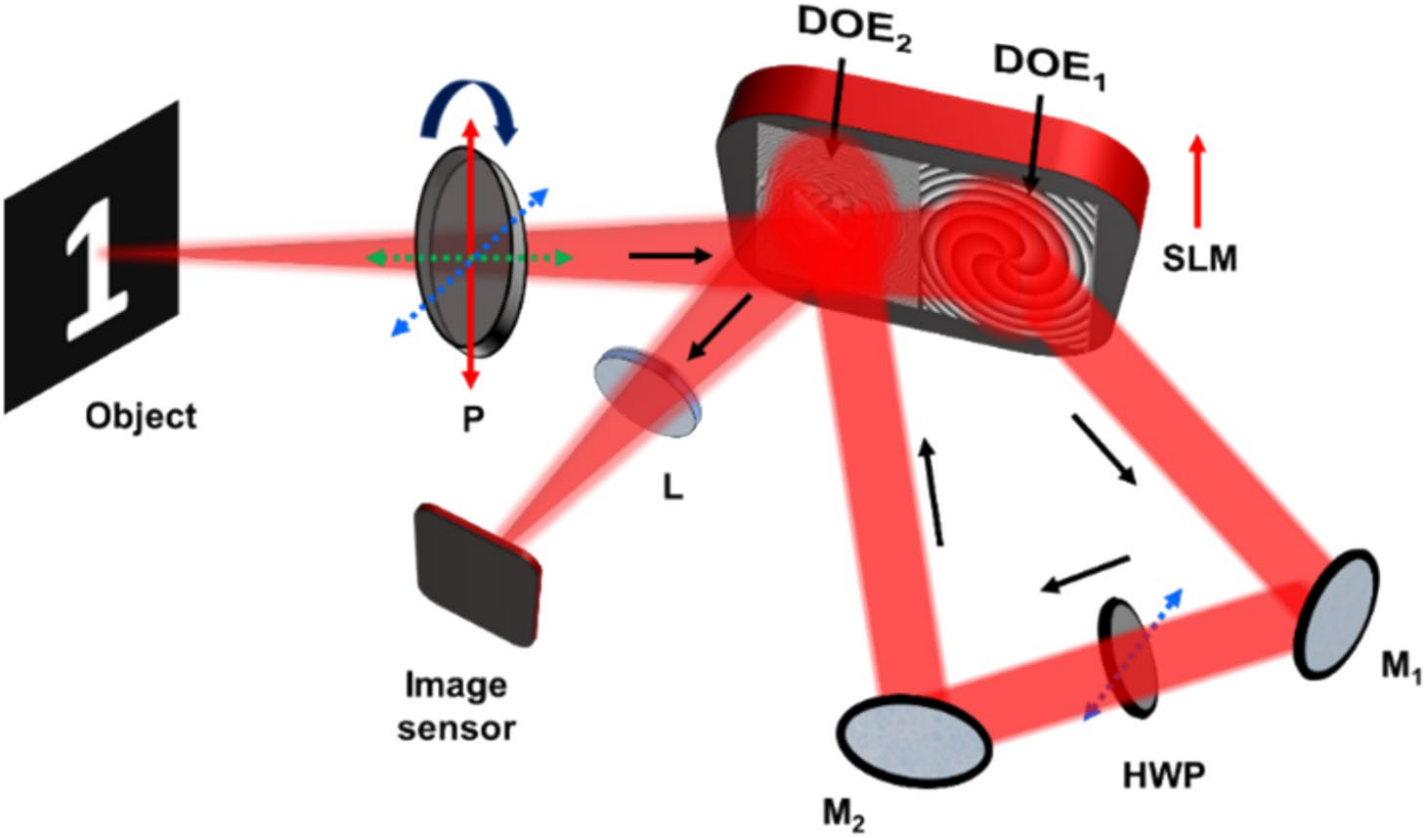
DOPP configuration using a single SLM. Two-headed arrows represent different polarization states. One-headed arrow represents the direction of the optical beam. [*DOE* diffractive optical element, *M* mirror, *SLM* spatial light modulator, *P* polarizer, *L* lens, *HWP* half-wave plate].

In^40–42^, polarization dependent blur was achieved either using two SLMs or using an SLM and a fabricated diffractive optical element^31^. In this study, polarization dependent blur has been achieved using a single SLM using the DOPP configuration as shown in (Fig. 2). In DOPP, two different DOEs are displayed at two halves of the SLM display to simultaneously achieve polarization-dependent dual-phase modulation. This technique leverages SLM’s polarization-dependent light modulation capability using two different DOEs. It enables recording polarization-discriminated object information in a single shot. A detailed description of DOPP configuration is provided in section “Experiments”.

## Simulation studies

**Fig. 3:**
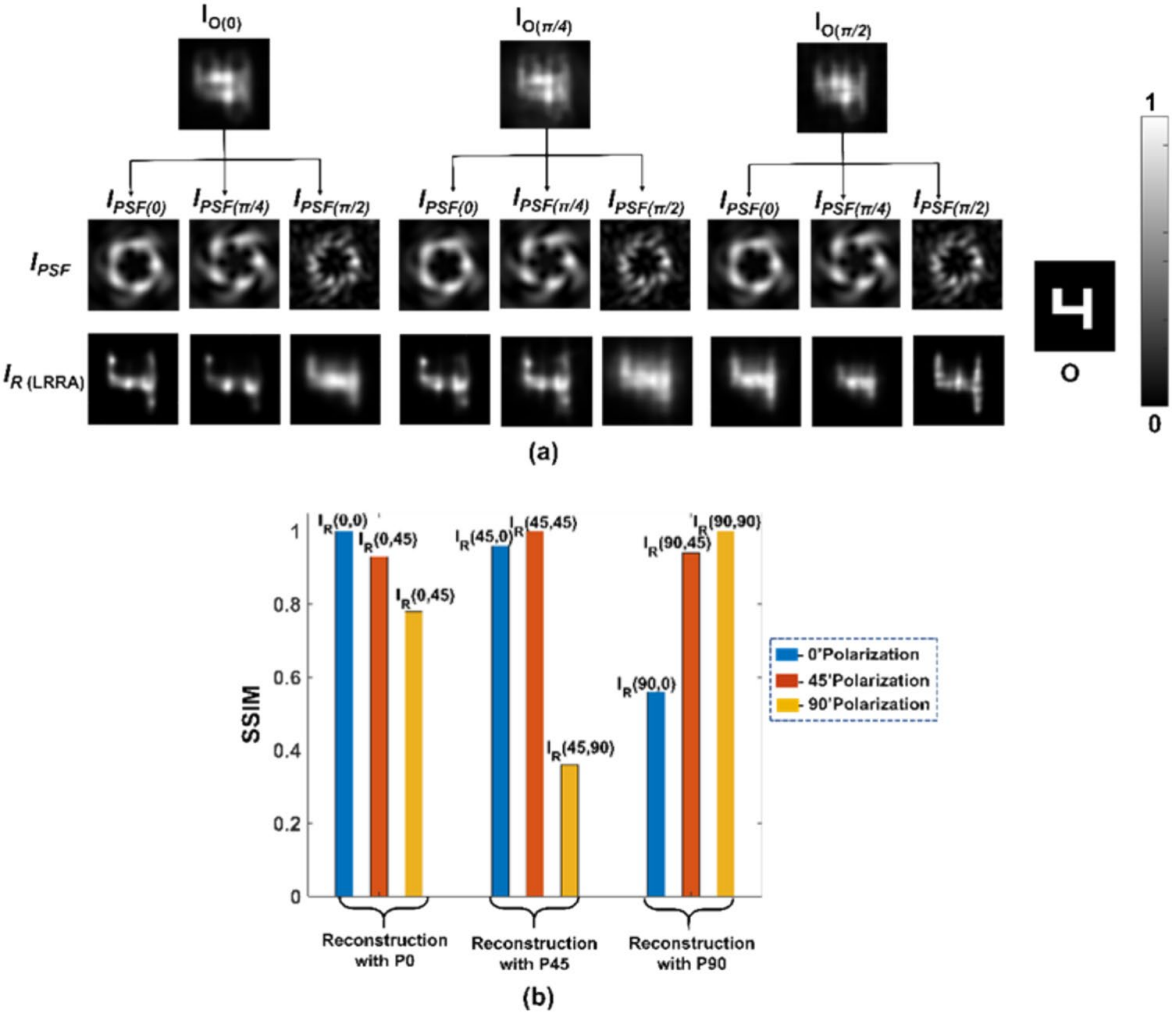
Simulation results of DOPP-CAI (**a**) reconstruction results, row1: object intensity distributions (*I*_*O*_) for O (digit ‘4’), row2: point spread functions (*I*_*PSF*_) at three polarization states, i.e., (φ = 0, π/4, and π/2), row3: Corresponding reconstruction results using LRRA. (**b**) SSIM index plot for reconstruction results for different *I*_*O*_ and *I*_*PSF*_ polarization combinations*.*

The simulation study of DOPP-CAI was carried out using MATLAB software. A matrix size of 2160 × 2160 pixels, wavelength λ = 660 nm with pixel size Δ = 3.8 µm was used for simulation of DOPP-CAI. A spiral lens of topological charge *L* = 5, mathematically written as, $\mathit{exp}\left[-i\pi ({\lambda f_1)}^{-1}{R}^{2}\right]\times exp\left(-iL\theta \right)$ where $R={\left({x}^{2}+{y}^{2}\right)}^{1/2}$ and a quasi-random diffractive lens (QRDL), given as $\mathit{exp}\left[-i\pi ({\lambda f_2)}^{-1}{R}^{2}\right]\times exp\left[i{\Phi }_{\sigma }\left(x,y\right)\right]$ where $f=40 cm$ and σ = 0.02 is the scattering degree of the quasi-random phase function $\phi$, are taken as DOE_1_ and DOE_2_, respectively, to demonstrate DOPP modulation. The design of QRDL is explained in supplementary material (S1). Digit ‘4’ was used as a test object to show polarization-dependent discrimination using DOPP-CAI. Figure 3a represents the simulation results of DOPP-CAI under the abovementioned conditions. In Fig. 3a, the first row and second row represent the simulated object intensity distributions (*I*_*O*_) for O (digit ‘4’) and point spread functions (*I*_*PSF*_) for three polarization states or SOP ( State of polarization ) which indicate the orientation and behavior of the electric field vector of light i.e., φ = 0, π/4, and π/2 respectively. The reconstruction results by processing *I*_*O*_ (φ = 0, π/4, and π/2) with *I*_*PSF*_ (φ = 0, π/4, and π/2) using LRRA deconvolution algorithm are shown in third row respectively. In the simulation studies, the optimal reconstructions were obtained for the following values: 10 ≤ *n* ≤ 20, 0.4 ≤ α ≤ 0.6, and β = 1. Details of LRRA are provided in supplementary material (S2).

Notably, polarization-dependent phase modulation can be observed in DOPP-CAI by intertwining the phase characteristics of two DOEs. Further, the simulation results were quantitatively assessed by calculating the Structured Similarity Index Measurement (SSIM) index for all reconstructed results, which is shown in (Fig. 3b). The highest SSIM value was obtained for the reconstruction results corresponding to *I*_*O*_ and *I*_*PSF*_, which were recorded at the same polarization state. Therefore, simulation results indicate better image recovery for the same SOP combinations than cross-SOP combinations of *I*_*O*_ and *I*_*PSF*_ and they also exhibit notable polarization-dependent blur.

## Experiments

**Fig. 4:**
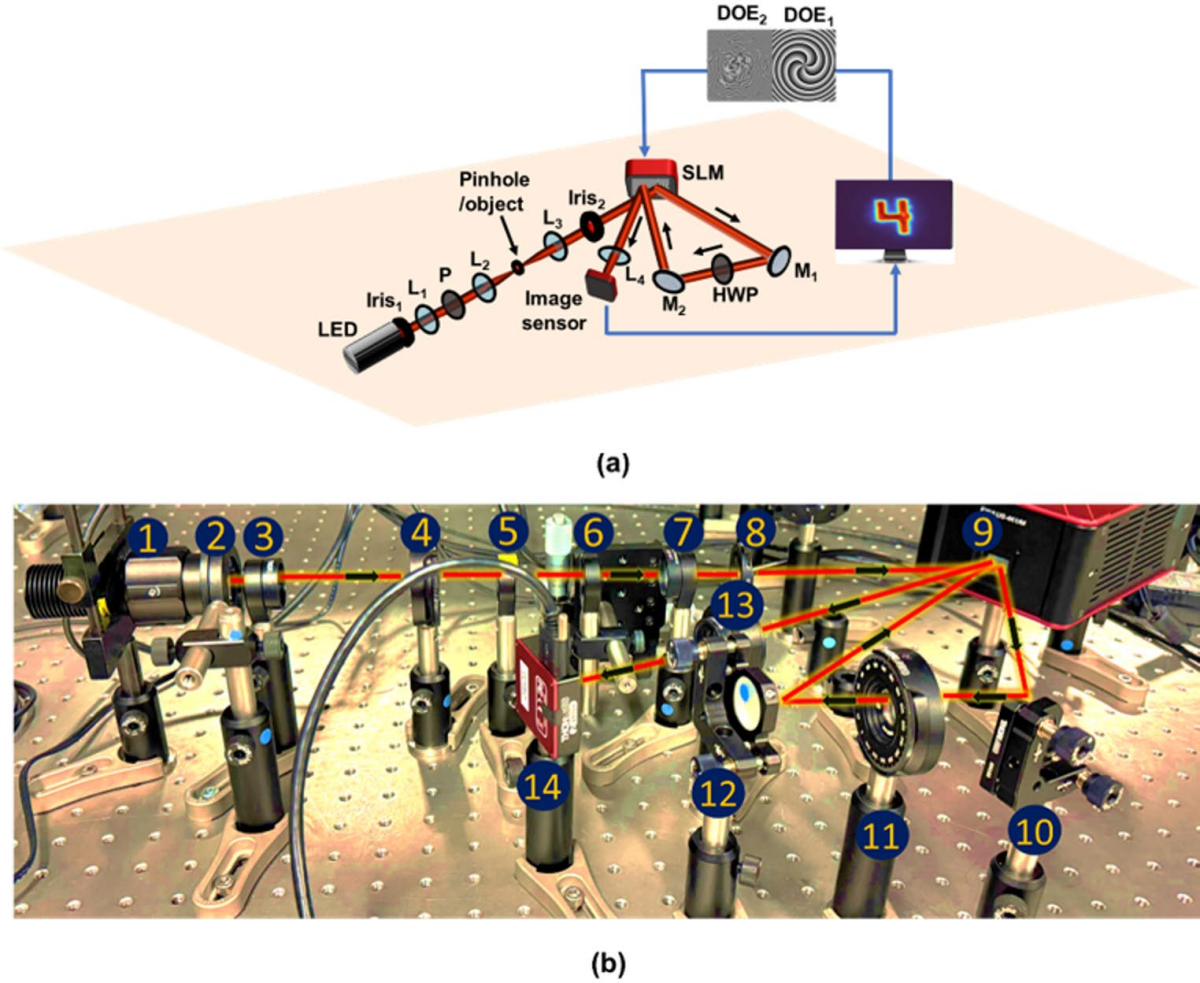
(**a**) Schematic of the experimental configuration of DOPP-CAI. (**b**) Photograph of tabletop experimental setup: (1) LED, (2) iris(I_1_), (3) refractive lens (L_1_), (4) polarizer, (5) refractive lens (L_2_), (6) object/pinhole, (7) refractive lens (L_3_), (8) iris (I_2_), (9) SLM, (10) Mirror(M_1_), (11) half wave plate (HWP), (12) Mirror(M_2_), (13) refractive lens (L_4_), (14) image sensor.

Figure 4a illustrates the schematic of the experimental setup of DOPP-CAI technique, and a photograph of the tabletop experimental setup is shown in (Fig. 4b). An incoherent red-light beam emanating from a high-power LED (λ = 660 nm and Δλ = 20 nm) was passed through a collimating lens (L_1_) of a focal length of 7.5 cm. The input light throughput was controlled using an iris. A linear polarizer (P_1_) was used to polarize the input light along φ = 0, π/4, and π/2. The polarization state ‘φ = 0’ refers to polarization along the active axis of SLM (H), polarization state ‘φ = π/2’ indicates that the polarization is orthogonal to the active axis of SLM (V), and polarization state ‘φ = π/4’ denotes the diagonal intermediate polarization state (D). The collimated light beam was focused on a pinhole/object using a refractive lens (L_2_) of focal length 5 cm to achieve critical illuminations. In this experiment, digit '4' from group-3 of R1DS1N—Negative 1951 USAF Test Target, Ø1″ is used as a test object (O). Corresponding point spread functions (*I*_*PSF*_*s*) were recorded using a pinhole of diameter 50 μm. Light from the pinhole/object was again collimated by a convex refractive lens (L_3_) of focal length of 5 cm and incident on the SLM ((Thorlabs Exulus-4K 1/M, 3840 × 2160 pixels, pixel size = 3.74 μm, Newton, MA, USA).) A dual-phase mask with two DOEs was displayed on the SLM by splitting its display into two halves. In this experiment, a spiral lens of topological charge *L* = 5 and a quasi-random diffractive lens (QRDL) are implemented as DOE_1_ and DOE_2_, respectively. An iris controls the beam size to ensure that it exposes only half of the active area of the SLM. The SLM is polarization sensitive and modulates only the light polarized along its active axis. The SLM is placed at a slightly tilted position from the optical axis to utilize the entire active area of the SLM display. After reflection from the first half of the SLM, the beam acquired the spiral phase of DOE_1_ in a H-polarization state, and this beam was redirected to the second half of the SLM using two plane mirrors (M_1_ and M_2_), respectively. To enable dual phase modulation of the SLM, a half-wave plate (HWP) was inserted between mirrors (M_1_ and M_2_), which interchanges the H and V polarization states and modulates the other component of the beam via the second modulation cycle of the SLM. For polarization states H and V, either DOE_1_ or DOE_2_ was activated, respectively. However, for polarization state D, both DOEs were activated. Therefore, polarization-discriminated dual phase modulation was achieved by rotating the linear polarizer (P_1_) at φ = 0, π/4, and π/2. The resultant light beam with dual phase modulation was directed to an image sensor (Zelux CS165MU/M 1.6 MP monochrome CMOS camera, 1440 × 1080 pixels with a pixel size of ~ 3.5 µm; Newton, MA, USA) using a bi-convex refractive lens (L_4_) of focal length 20 cm to record the object intensity distributions (*I*_*O*_) and corresponding point spread function (*I*_*PSF*_) for different planes and wavelengths respectively. The approach described herein enables polarization-discriminated dual-phase control using a single SLM. Hence, multiple SLMs and additional polarization components are not required.

DOPP-CAI was experimentally demonstrated in three steps. In the first step, a point spread function library for three polarization states (φ = 0, π/4, and π/2), at *z*_*s*_ = 5 cm was recorded with an image sensor (Zelux CS165MU/M 1.6 MP monochrome CMOS camera, 1440 × 1080 pixels with a pixel size of ~ 3.5 µm; Newton, MA, USA). In the next step, the object intensity distributions (*I*_*O*_) of object ‘digit 4’ were recorded for three polarization states (φ = 0, π/4, and π/2), at *z*_*s*_ = 5 cm, respectively. In the final step, polarization-discriminated object information was reconstructed by correlating the PSF library with the recorded object intensity distributions using LRRA ^25^.

## Results and discussion

**Fig. 5:**
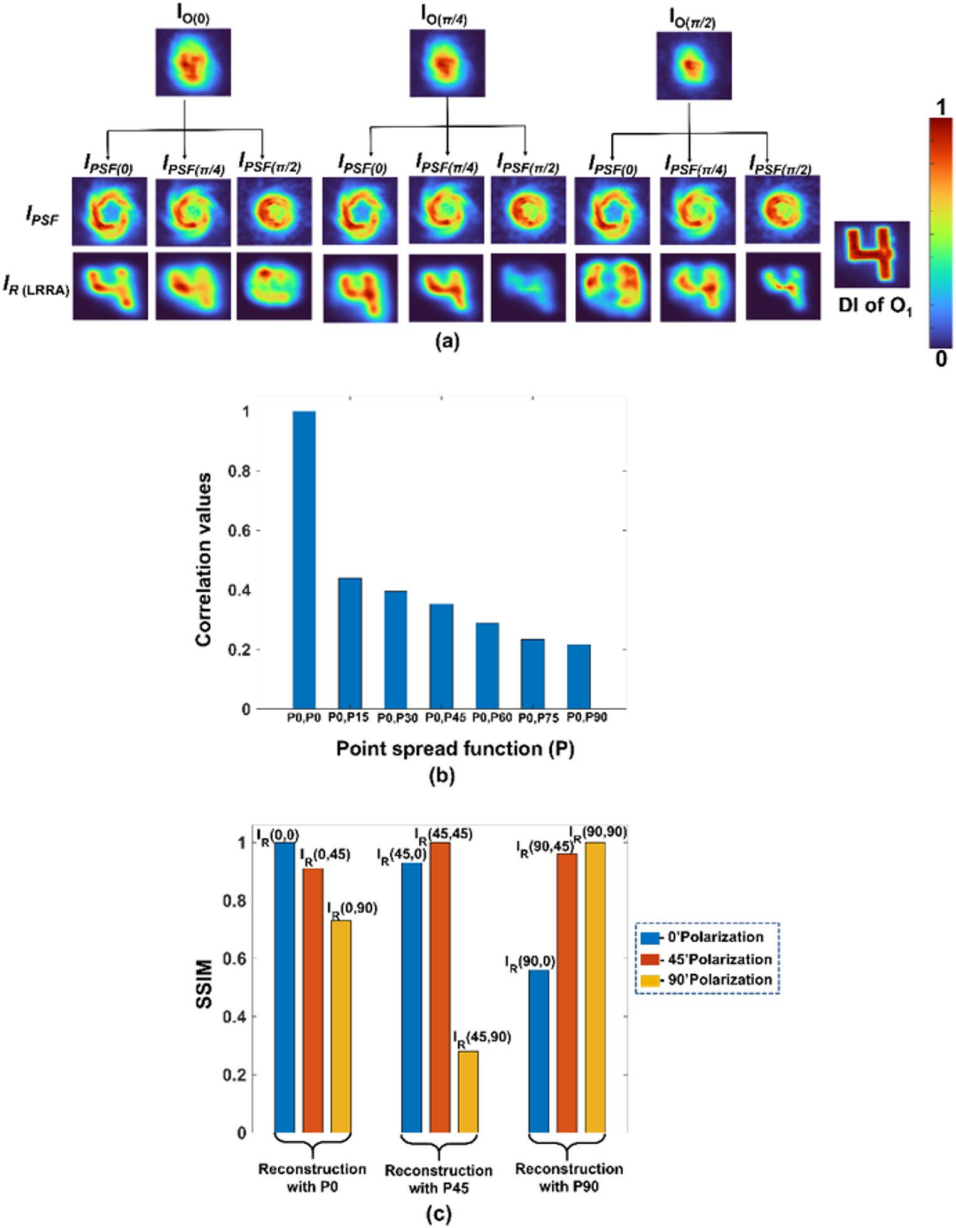
Experiment results of DOPP-CAI. (**a**) Reconstruction results (*z*_*s*_ = 5 cm), row1: *I*_*O*_ for O (digit ‘4’), row2: *I*_*PSF*_s at three polarization states, i.e., H (φ = 0), D (φ = π/4), and V (φ = π/2), row3: Corresponding reconstruction results using LRRA. (**b**) *I*_*PSF*_ correlation plot for different polarization combinations. (**c**) SSIM values plot for reconstruction results for different *I*_*O*_ and *I*_*PSF*_ polarization combinations.

Experimental results of DOPP-CAI are shown in (Fig. 5). In Fig. 5a, the first row and second row represent the recorded *I*_*O*_s for O (digit ‘4’) and point *I*_*PSF*_s at *z*_*s*_ = 5 cm at three polarization states, i.e., H (φ = 0), D (φ = π/4), and V (φ = π/2) for red wavelength (λ = 660 nm). Corresponding reconstruction results using LRRA are arranged in row 3. It should be noted that for polarization state H (φ = 0), only DOE_1_ (spiral lens) is active due to first phase modulation of SLM, and only DOE_2_ (QRDL) is active for V (φ = π/2) due to second phase modulation of SLM. However, polarization state D (φ = π/4) is an interesting case, as both DOEs are active and represent SLM’s dual phase modulation state.

A significant blur is observed for different polarization states. Therefore, this approach offers polarization-dependent phase switchability in DOPP-CAI. The reconstruction results exhibit a significant blur while switching the polarization state from H (φ = 0) to V (φ = π/2). In the experimental studies, the reconstruction parameters in the following ranges 20 ≤ *n* ≤ 60, 0.2 ≤ α ≤ 0.6, and β = 1 gave the optimal results. It is observed that the best reconstruction is obtained for *I*_*PSF*_ and *I*_*O*_ at the same polarization state (1st element (0,0), 5th element (π/4, π/4), and 9th element (π/2, π/2) of row 3) as compared to cross-polarization states ((0, π/4), (0, π/2), (π/4, 0), (π/4, π/2), ((π/2, 0), (π/2, π/4)) respectively. The polarization cross-talk is quantified using two parameters namely cross-correlation by a phase-only filter and SSIM. A bar plot for the correlation value at (*x* = 0,*y* = 0) between a reference *I*_*PSF*_(φ = 0) with *I*_*PSF*_s(φ = 0, 15, 30, 45, 60, 90) is shown in (Fig. 5b). When φ was changed from 0 degrees, there is a significant drop of correlation value but after that the variation is linear with smaller changes compared to the first change for 15 degrees. The highest and lowest correlation values are obtained for (P0, P0) and (P0, P90), respectively, while it follows a decreasing trend for intermediate PSF combinations. Therefore, it can be concluded that the best reconstruction results for the same polarization state combinations are due to the higher correlation values than cross-polarization combinations. The feasibility of experimental results is validated by calculating the SSIM values of reconstruction results for different *I*_*O*_ and *I*_*PSF*_ polarization combinations. It is observed that the reconstruction results corresponding to *I*_*O*_ and *I*_*PSF*_ for the same polarization states (*I*_*R*_(0,0), *I*_*R*_(45,45), and *I*_*R*_(90,90)) yield the highest SSIM value, while lower values of SSIM were obtained for reconstruction results for different polarization states. Further, the polarization resolution ability (PRA) of DOPP-CAI system is quantified as the average percentage change of normalized intensity correlation per radian given as (ΔI_c_% /Δφ). From Fig. 5b, we observed that the normalized intensity correlation decreases from 0.44 at (P0,P15) to 0.2161 at (P0,P90) giving a percentage drop of 50.89% over a polarization angle change of 1.309 radians (75⁰),the PRA can be obtained as 38.88% per radian. This demonstrates the system’s strong ability to distinguish polarization changes.

## Conclusion and future perspectives

In this study, a new CAI technique, DOPP-CAI, is proposed and experimentally demonstrated for three polarization states (φ = 0, π/4, and π/2). We propose a modified dual-phase modulation approach using a single SLM by displaying two different DOEs at different halves of the SLM display. Unlike the conventional dual-phase modulation approach, it does not require multiple SLMs and additional polarization components. This technique leverages the polarization-dependent light modulation capability of SLM using two different DOEs. It enables recording polarization-discriminated object information in a single shot, and the corresponding object information is reconstructed using the LRRA algorithm. Sharp reconstruction results are achieved for cases with same polarization states and blurred reconstructions were obtained for cases with different polarization states, owing to the strong dependency of correlation value on polarization orientation. Polarization-discriminated blur in reconstruction results indicates the possibilities of improved imaging characteristics in coded aperture imaging. The spatial resolutions of the DOPP-CAI system, namely lateral and axial resolutions are given as ~ λ/NA and ~ λ/NA^2^ respectively like any microscope. Therefore, for λ = 660 nm, NA = 0.1, the lateral and axial resolutions are ~ 10 μm and 85 μm, respectively. The current setup supports single-shot acquisition, and therefore the imaging speed is given by the camera frame rate which is 34.8 fps for Zelux CS165MU/M 1.6 MP monochrome CMOS camera, 1440 × 1080 pixels with a pixel size of ~ 3.5 µm; Newton, MA, USA. The developed DOPP-CAI can be extended to spectrum and depth, enabling upto 6D imaging. The preliminary results of 4D-DOPP-CAI along 3D space, and polarization are discussed in the Supplementary Material (Section S3). In 4D-DOPP-CAI, the displacement of two objects across two axial planes was used to mimic 3D imaging for a thick material. Enabling depth-resolved reconstruction, DOPP-CAI successfully illustrated the system’s capability to recover polarization-dependent 3D information. However, the reconstruction quality in 4D-DOPP-CAI still needs improvement. The possible directions are optimizing signal-to-noise ratio (SNR) and field of view (FOV). Addressing such issues in DOPP-CAI can lead to 4D imaging of real objects in the future. We believe the proposed compact and robust experimental scheme for polarization-dependent dual-phase modulation can benefit the development of advanced imaging systems.

## Supplementary Information

Supplementary Information.

## Data Availability

The datasets used and/or analyzed during the current study are available from the corresponding author upon reasonable request.
