# Supplementary material for: Single shot polarization resolved coded aperture imaging
Source: Sci Rep. 2025 Jul 2;15:23164. doi: 10.1038/s41598-025-04657-2 (PMC12223030; doi:10.1038/s41598-025-04657-2)
Supplement: Supplementary file 1 — Supplementary Information. [file 41598_2025_4657_MOESM1_ESM.docx]

**Supplementary materials for “Single Shot Polarization Resolved Coded Aperture Imaging”**

Narmada Joshi,^1, *, Ϯ^ Vipin Tiwari,^1, Ϯ^ Aile Tamm,^1^ Joseph Rosen,^2^ and Vijayakumar Anand^1,3^

^1^Institute of Physics, University of Tartu, W. Ostwaldi 1, 50411 Tartu, Estonia

^2^School of Electrical and Computer Engineering, Ben Gurion University of the Negev, P.O. Box 653, Beer-Sheva 8410501, Israel

^3^Optical Sciences Center Swinburne University of Technology, Hawthorn, Melbourne, VIC 3122, Australia

* Corresponding author [narmada@ut.ee](mailto:narmada@ut.ee)

^Ϯ^ *Authors contributed equally to the manuscript***.**

**S1:** **Design of quasi-random diffractive lens (QRDL)**

**
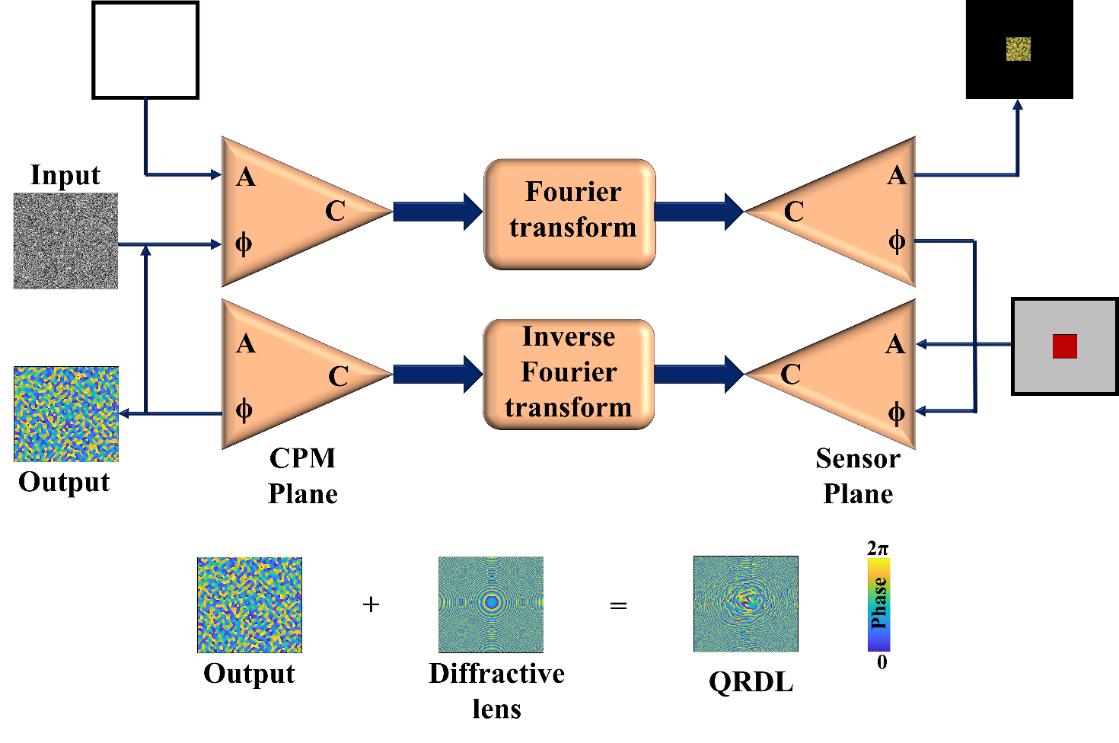
**

**Figure S2.** Schematic of the GSA for the design of QRDL.

Quasi-random diffractive lens (QRDL) is a specially designed DOE, whose phase distribution can be expressed as $exp\left( {j\Phi}_{QRL} \right)=\exp\left[ -i\pi({\lambda f_{2})}^{-1}R^{2} \right]\times exp\left( -i\Phi_{r} \right)$, where $\Phi_{r}$ is a 2D quasi-random distribution [0, 2π] whose scattering ratio σ can be controlled by the Gerchberg-Saxton algorithm (GSA) by applying a limited support mask constraint in the spectral domain. The schematic of QRDL is shown in Figure S1. The Fourier-based GSA is used to design the QRDL. Two planes of interest are namely CPM plane and sensor plane. Sensor plane was located at the Fourier plane of the CPM plane. To Obtain pure phase function in the CPM plane with a uniform magnitude over a desired area, the phase constraint is applied at the CPM plane while keeping the amplitude fixed at one. The amplitude is constrained within a specific region at the sensor plane, and the phase is iterated accordingly. The process involves alternating forward, and inverse Fourier transforms to synthesize the complex amplitudes at both planes. A phase pattern at the CPM plane becomes stable after about few iterations.

**S2:** **Lucy-Richardson Rosen Algorithm (LRRA)**

The Lucy-Richardson-Rosen algorithm (LRRA) has been developed by combining two well-established deconvolution algorithms, i.e., non-linear reconstruction (NLR) [1] and Lucy-Richardson algorithm (LRA) [2,3]. In NLR, the reconstructed image is optimized by tuning two non-linear parameters, i.e., $\alpha\mathrm{and} \beta$ between -1 and 1 to obtain minimum entropy. On the other hand, LRA uses an iterative approach, where the reconstructed image is iterated in a loop until an optimal reconstruction is achieved. LRRA leverages the combined approach of NLR and LRA, yielding a better estimation of reconstructed images with rapid convergence [4].

In LRRA, the reconstructed image is given as

$I_{R}(p)=I_{R}(p-1)\left[ \frac{I_{O}}{I_{R}(p-1)\bigotimes I_{PSF}}*_{\beta}^{\alpha}I_{PSF} \right]$ (S1)

where ‘$*_{\beta}^{\alpha}$’ is the non-linear correlation operator given as ${u*}_{\beta}^{\alpha}v=\mathcal{F}^{-1}\left\{ \left| U \right|^{\alpha}\left| V \right|^{\beta}\exp\left( i\Phi_{U} \right)\cdot exp\left( -i\Phi_{V} \right) \right\}$, where *U* and *V* are the Fourier transforms of *u* and *v* respectively. $p$ is the number of iterations. The schematic (flowchart) of LRRA is shown in the Figure S2.


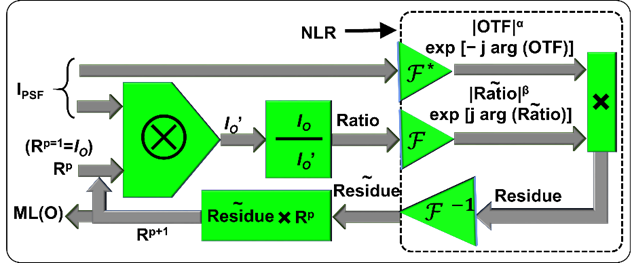


**Figure S2.** Schematic of the Lucy‒Richardson–Rosen algorithm. OTF – optical transfer function; *p*—number of iterations of LRRA; ⊗—2D convolutional operator; *R^p^* is the *p*^th^ solution; and *p* is an integer; when *p* = 1, *R^p^* = $I_{O}$; and *α* and *β* are varied from −1 to 1; ~ Fourier transform; ‘*’ complex conjugate; NLR is a nonlinear reconstruction; and $\mathcal{F}$ and $\mathcal{F}^{-1}$ are Fourier and inverse Fourier transforms, respectively.

**S3.** **Four-dimensional (4-D) DOPP-CAI**

**S3.1 Simulation studies**

The four-dimensional imaging capabilities of DOPP-CAI are simulated using MATLAB software. A matrix size of 2160 × 2160 pixels, wavelength λ = 660 nm with pixel size Δ = 3.8 µm, Δz= 0.5 m was used for simulation of 4D-DOPP-CAI. A spiral lens of topological charge (*l*) = 5 , mathematically written as, $\exp\left[ -i\pi({\lambda f)}^{-1}R^{2} \right]\times exp\left( -iL\theta\right)$ where $R=\left( x^{2}+y^{2} \right)^{1/2}$ and a quasi-random diffractive lens (QRDL), given as $\exp\left[ -i\pi({\lambda f)}^{-1}R^{2} \right]\times exp\left[ i\Phi_{\sigma}\left( x,y \right) \right]$, (where σ=0.2 is the scattering degree of the quasi-random phase function $\phi$ and *f*=40 cm), are taken as DOE_1_ and DOE_2_, respectively. In 4D- DOPP-CAI, two test objects, i.e., digit ‘4’ (O_1_) and digit ‘1’ (O_2_), were used to show polarization-dependent discrimination using DOPP-CAI in two planes with axial separation of Δz= 0.5 m. In 4D DOPP-CAI, I_O_ at three polarization states (φ = 0, π/4, and π/2) and two depths (Δz= 0.5 m) were stitched computationally into a single matrix of order 3**×** 2 and reconstructed from I_PSF_ at two planes and three polarization states, respectively.


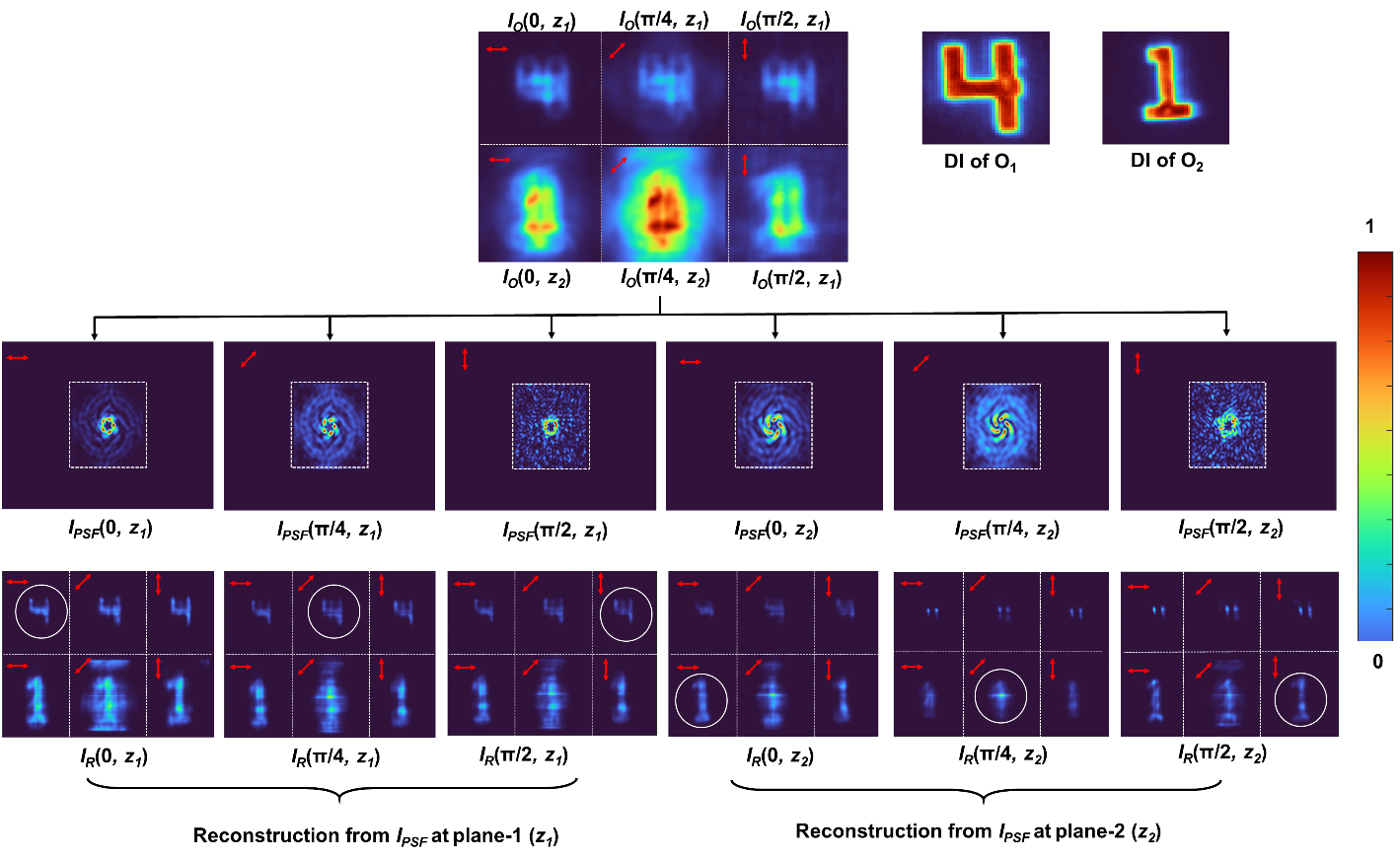


Figure S3. Simulation results of 4D-DOPP-CAI. Row-1: The stitched matrix (*I_O_*) of order 3× 2 and direct images (DI) of O_1_ and O_2_ . Row-2: simulated *I_PSF_* for three polarization states, i.e., φ = 0, π/4, and π/2 at plane-1 (first 3 elements) and plane-2 (last 3 elements). Row-3: The reconstruction results by processing *I_O_* (φ = 0, π/4, and π/2) with *I_PSF_* (φ = 0, π/4, and π/2) using LRRA deconvolution algorithm. Red arrows represent three polarization states (φ = 0, π/4, and π/2). Square window with white outline in row-2 illustrates the region of interest (ROI) of *I_PSF._* The white circle in row-3 elements highlights the sharpest reconstruction from *I_O_* and *I_PSF_* at the same polarization states.

Figure S3 represents the simulation results of 4D-DOPP-CAI under the abovementioned conditions. In the first row of Figure S3, the stitched matrix (I_O_) of order 3**×** 2 and direct images (DI) of O_1_ and O_2_ are shown respectively. The second row represents the simulated *I_PSF_* for three polarization states, i.e., φ = 0, π/4, and π/2 at plane-1 (first 3 elements) and plane-2 (last 3 elements), respectively. The reconstruction results by processing *I_O_* (φ = 0, π/4, and π/2) with *I_PSF_* (φ = 0, π/4, and π/2) using LRRA deconvolution algorithm are shown in row-3. The image recovery with *I_PSF_* of different depths causes significant blurring at different polarization states. For example, the reconstruction of O_1_ in the first three elements of row 3 is sharper while reconstructing from *I_PSF_*  at plane-1. On the other hand, next three elements of row-3 exhibit sharper recovery of O_2_ from *I_PSF_*  at plane-2. Moreover, sub-elements of the reconstruction matrix (*I_R_*) demonstrate the polarization-dependent blur in 4D-DOPP-CAI at three polarization states, i.e., φ = 0, π/4, and π/2, where sharpest reconstruction is observed for *I_O_* and *I_PSF_* pair at same polarization state.

**S3.2 Experiment**

The four-dimensional (3D space and polarization) DOPP-CAI was experimentally demonstrated using DOPP experimental setup (Figure 4 of main article). In the first step, a 4D-*I_PSF_* library is recorded at two planes (axial separation of Δz= 0.5 cm) and three polarization states (ϕ=0, π/4, π/2) using a pinhole of diameter 50 μm. Digit '4' and digit ‘1’ from group-3 of R1DS1N—Negative 1951 USAF Test Target, Ø1” were used as test objects O_1_ and O_2_ to record 4D object information (*I_O_*) at two planes (axial separation of Δz= 0.5 cm) and three polarization states (ϕ=0, π/4, π/2) respectively. Similar to simulation studies, I_O_ at three polarization states (φ = 0, π/4, and π/2) and two depths (Δz= 0.5 cm) were stitched computationally into a single matrix of order 3**×** 2 and reconstructed from I_PSF_ at two planes and three polarization states, respectively.


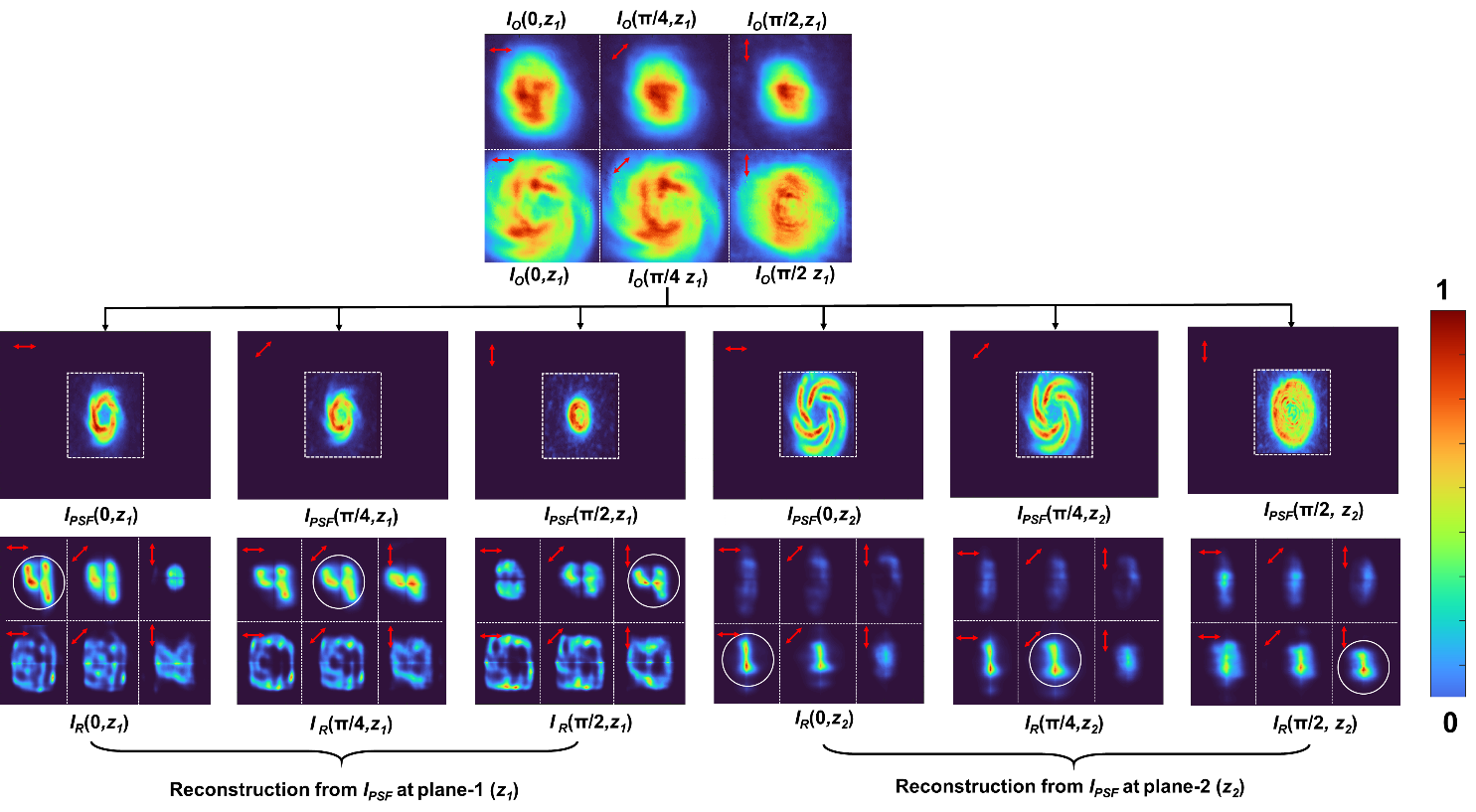


Figure S4. Experimental results of 4D-DOPP-CAI. Row-1: The stitched matrix (*I_O_*) of order 3× 2 and direct images (DI) of O_1_ and O_2_ . Row-2: recorded *I_PSF_* for three polarization states, i.e., φ = 0, π/4, and π/2 at plane-1 (first 3 elements) and plane-2 (last 3 elements). Row-3: The reconstruction results by processing *I_O_* (φ = 0, π/4, and π/2) with *I_PSF_* (φ = 0, π/4, and π/2) using LRRA deconvolution algorithm. Red arrows represent three polarization states (φ = 0, π/4, and π/2). Square window with white outline in row-2 illustrates the region of interest (ROI) of *I_PSF._* The white circle in row-3 elements highlights the sharpest reconstruction from *I_O_* and *I_PSF_* at the same polarization states.

The experimental results of 4D-DOPPP-CAI are presented in three rows in Figure S4. The first row shows the computationally stitched matrix of order 3×2, containing 4D object intensity distribution (*I_O_*) at three polarization states (ϕ=0, π/4, π/2) and two depths (Δz= 0.5 cm). In the second row, the *I_PSF_* recorded at three polarization states, i.e., φ = 0, π/4, and π/2 at plane-1 (first 3 elements) and plane-2 (last 3 elements), are presented, respectively. The third row depicts the reconstruction results obtained using the LRRA algorithm. The results demonstrate that sharper object image reconstruction using *I_PSF_* corresponds to the same polarization states and has a significant blur concerning non-matching polarization states. Therefore, 4D imaging capabilities of DOPP-CAI was demonstrated using simulation and experiments.

**S4. MATLAB codes**

**S4.1 MATLAB code for LRRA**

PSF=’load PSF’;

O=’load Object’;

fn = O; % at the first iteration

OTF = psf2otf(PSF,size(O));

iterations =x; % x is the number of iterations

figure; colormap turbo

for i=1:iterations

i

ffn = fft2(fn);

alpha = 1;

beta = 1;

Hfn = ((abs(OTF).^alpha).*exp(1i*angle(OTF))).*((abs(ffn).^beta).*exp(1i*angle(ffn)));

iHfn = ifft2(Hfn);

ratio = O./iHfn;

iratio = fft2(ratio);

alpha = 0.6; % $0\leq alpha\leq1$

beta =1; % $-1\leq beta\leq1$

res =conj ((abs(OTF).^alpha).*exp(1i*angle(OTF))).*((abs(iratio).^beta).*exp(1i*angle(iratio)));

ires = ifft2(res);

fn = ires.*fn;

% fn=fn.^2;

imagesc(abs(fn).^1);

pause(0.1)

end

result = abs(fn);

**S4.2 MATLAB code for QRDL design**

z1=1000;

z2=0.4;

Q1=exp(1i*(pi/(lambda1*z1))*(X.*X+Y.*Y));

Q2=exp(1i*(pi/(lambda1*z2))*(X1.*X1+Y1.*Y1));

Matrix=2*pi*rand(N,N);

Sensor1=zeros(N,N);

Sensor1(1060:1100,1060:1100)=1;

iter=1;

for p=1:iter

A1=angle(fft2(exp(1i*Matrix)));

A2=Sensor1.*exp(1i*A1);

Matrix=angle(ifft2(A2));

end

B1=Matrix;

f=0.4;

RL=exp(-1i*(pi/(lambda1*(f)))*(X.*X+Y.*Y)).*exp(1i*Matrix);

Lens1=(angle(RL)+pi)/(2*pi);%Random lens

imagesc(angle(RL));

**References**

1. Rai MR, Vijayakumar A, Rosen J. Non-linear adaptive three-dimensional imaging with interferenceless coded aperture correlation holography (I-COACH). Opt Express 26, 18143–18154 (2018).
2. Richardson WH, Bayesian-based iterative method of image restoration, J. Opt. Soc. Am, 62, 55-59 (1972).
3. Lucy LB, An iterative technique for the rectification of observed distributions, Astron J, 79, 745 (1974).
4. V. Anand, M. Han, J. Maksimovic, S. H. Ng, T. Katkus, A. Klein, K. Bambery, M. J. Tobin, J. Vongsvivut, and S. Juodkazis, “Single-shot mid-infrared incoherent holography using Lucy-Richardson-Rosen algorithm,” Opto-Electron. Sci. 1, 210006 (2022).
5. Gerchberg R. W, Saxton W. O. A practical algorithm for the determination of phase from image and diffraction plane pictures. Optik, 35, 227–46 (1972).
